# Which patients do I treat? An experimental study with economists and physicians

**DOI:** 10.1186/2191-1991-2-1

**Published:** 2012-01-05

**Authors:** Marlies Ahlert, Stefan Felder, Bodo Vogt

**Affiliations:** 1 Faculty of Law, Economics and Business, Martin-Luther-University Halle-Wittenberg, (Große Steinstraße 73), (0108) Halle an der Saale, Germany; 2 Faculty of Business and Economics, University of Basel, (Peter Merian-Weg 6), (4002) Basel, Switzerland and Faculty of Economics and Business Administration, Duisburg-Essen University, (Univer-sitätsstraße 12), (45117) Essen, Germany; 3 Faculty of Economics and Management, Otto-von-Guericke-University Magdeburg, (Univer-sitätsplatz 2), (39106) Magdeburg, Germany

**Keywords:** experimental economics, social orientation, individual choices, allocation of medical resources, principles of distribution

## Abstract

This experiment investigates decisions made by prospective economists and physicians in an allocation problem which can be framed either medically or neutrally. The potential recipients differ with respect to their minimum needs as well as to how much they benefit from a treatment. We classify the allocators as either 'selfish', 'Rawlsian', or 'maximizing the number of recipients'. Economists tend to maximize their own payoff, whereas the physicians' choices are more in line with maximizing the number of recipients and with Rawlsianism. Regarding the framing, we observe that professional norms surface more clearly in familiar settings. Finally, we scrutinize how the probability of being served and the allocated quantity depend on a recipient's characteristics as well as on the allocator type.

JEL *Classification*: A13, I19, C91, C72

## 1 Introduction

Prioritizing medical services and redefining access to health care are high on political agendas across the globe. Several countries have appointed commissions to define the rules for the health technology assessments and cost-benefit analyses which guide allocation decisions in health care. Experts on such panels, in particular health economists and health ethicists, tend to ignore the fact that not all medical allocation decisions can be made on the level of fixing general rules. If that were feasible, trade-off decisions behind a veil of uncertainty would involve only statistical lives. In fact, the allocation of scarce medical resources and the pursuant withholding of care cannot always be 'pre-programmed' by general rules. Not only will the individuals from whom care must be withheld have a face and an identity, the allocator himself will be a specific individual who will have to make an allocation choice in a specific situation. Consequently, specific individuals are affected by decisions over which the allocator has discretionary powers. These within rule-choices (as opposed to the choice of rules) are not determined by the rules. They must be made by an allocator according to his judgment.

It is therefore important to analyze which allocation is chosen under which circumstances, and, in particular, to evaluate how medical care is allocated in the conflict between efficiency, selfish behavior, and the social orientation of decision makers. The experimental method has proven useful for testing theories on economic allocation. In particular, fairness ideals have been extensively scrutinized in the experimental laboratory (for a recent study see [1]). However, to our knowledge no experimental test has been carried out in the medical setting yet. We model the medical allocation problem and experimentally test the power of several theoretical concepts (ranging from utilitarianism to Rawlsian behavior) to predict subjects' choice behavior.

The goal of this paper is to study allocation decisions by prospective physicians and econo-mists. The experiment is designed to reveal when and how individuals deviate from the self-regarding preferences induced by the embedded monetary reward function. Will strict payoff maximizers - individuals who conform to the preferences induced by the reward function - prevail, or will we find deviations from such behavior that signify other relevant influences on the process of passing judgment? Are the choices made more in line with utilitarian principles or with an egalitarian rule a)? Do the principles applied depend on the framing of the problem, and do economists decide differently than physicians?

The paper is organized as follows: Section 2 presents the allocation problem and the solutions for four different types of allocation rules. Based on these rules, we characterize four classes of allocators: two utilitarian-oriented types (social utilitarian and purely selfish) and two types leaning towards egalitarianism (Rawlsian and maximizing the number of recipients). Section 3 describes details of the experimental design, including the characteristics of the potential recipients. We also calculate and compare the payoffs for ideal types of the four classes of allocators. In section 4, we classify the subjects who participated in the experiment based on their choices. We study the effects that arise from framing the allocation problem in a neutral and a medical fashion, where the allocator is described as a physician and the potential recipients as patients. Moreover, we compare the choices made by economists and physicians. In section 5, we investigate how the choices of different types of allocators depend on the minimum needs and productivity of potential recipients. In order to find out which subgroup of recipients is served and how much they receive, we first analyze the determinants of a positive recipient payoff using a logit model. Then we use ordinary least squares regression to analyze the determinants of the size of a recipient's payoff, conditional on it being positive. Section 6 discusses and summarizes our findings.

## 2 The allocation problem and possible solutions

In our experiment, an allocator distributes a resource among seven potential recipients. The individual recipients each require a specific minimum quantity of the resource in order to achieve a positive payoff. The potential recipients also vary in their productivity at transforming the quantity they receive into a payoff for themselves. The allocator's payoff is a function of the sum of the recipients' payoffs. Moreover, the allocator faces a fixed fine for each individual he fails to serve (i.e. the individual does not receive his minimum quantity nor, therefore, a payoff). We do not set out to test the validity of the assumed other-regarding concerns in this paper. Such motives, however, appear to be prevalent in common medical allocation situations b).

While a payoff maximizing allocator earns a maximal profit, an allocator following a rule not dictated by the preferences induced by the payoff function - for instance an egalitarian rule - loses out on profits. The experiment thus sheds light on the classic equity-efficiency tradeoff in a setting in which efficiency is not judged against purely selfish motives but relative to a complex evaluation.

More specifically, the allocator (individual 0) allocates ration *r_i _*to *n *individuals (*i *= 1, 2,..,*n*). With the endowment given by *R*, the allocator's choice is restricted by $\underset{i}{\sum }r_{i}\leq R$. The potential recipients are characterized by two parameters, *m_i _*and *p_i_*. *m_i _*is the minimum ration an individual needs to obtain a positive payoff, while *p_i _*is a productivity factor, transforming the allocated ration into a payoff for the recipient. In the medical setting, *m_i _*represents a physician's minimal time or effort required to treat the patient and *p_i _*stands for the probability of treatment success or the effectivity of the treatment. The payoff of individual *i *= 1, 2,..,*n *is then

(2) $$\pi_{i}=\left\{\begin{array}{c} 0,\text{ if }r_{i}<m_{i}\\ r_{i}\cdot p_{i}\text{ if }r_{i}\geq m_{i} \end{array}\right).$$

The allocator incurs a fine equal to *c *for every individual who does not receive the minimum ration m*_i _*c). In the medical setting, *c *corresponds to the physician's disutility of not treating a patient. One might interpret this as other-regarding preferences, typically due to empathy or internalized professional norms d). *c *is the same for all recipients who are not served and patients who are not treated.

Finally, the allocator participates in the recipients' payoffs with the factor *t*. This design feature introduces the second element of other-regarding preferences on the part of the allocator. The allocator's payoff *π*_0 _is *t *times the sum of the recipients' payoffs, minus all fines e):

(3) $$\pi_{0}=t\cdot \underset{i}{\sum }\pi_{i}-\underset{i|\pi_{i}=0}{\sum }c.$$

### 2.1 The own payoff maximizing allocator and the social utilitarian allocator

The own payoff maximizing allocator OPMA maximizes a target function

(4) $$W_{OPMA}\left(\pi_{0},\pi_{1},\pi_{2},...,\pi_{n}\right)=\pi_{0},$$

where *π*_0 _is determined according to (3). His optimal choice can be characterized as follows: He first ranks the individuals in decreasing order of their productivity factor *p_i_*, and then individuals with equal productivity in increasing order of their minimum required amount *m_i_*. Let *K *be the ranked set of possible recipients, with *k *= 1 as the most productive individual with the smallest *m_i_*, (or one of them, if there are several). The allocator will serve *k *= 1 first, provided that *m*_1 _≤ *R*. His remaining endowment then amounts to *R*-*m*_1_. Secondly, in consecutive order starting with *k *= 2, he will compare each individual to *k *= 1 and perform the following dominance test:

(5) $$t\cdot m_{k}\cdot p_{k}+c>\;t\cdot m_{k}\cdot p_{1}?$$

The test calculates the opportunity costs of allocating *m_k _*to the most productive individual. It consists of the foregone revenue *t*·*m_k_*·*p_k _*and the fine *c*. If the opportunity costs are larger than the revenue from allocating *m_k _*to *k *= 1 (i.e. a positive test outcome), the OPMA will serve *k *to the extent permitted by the remaining endowment. This procedure is continued along the ranked set *K*, spending *m_k _*if the individual *k *fulfills the test, and stops once the remaining endowment is too small to serve a further individual. The allocator will then give the remainder to the most productive individual, since this yields the maximal additional payoff. Note that if the fine were zero, no individual except *k *= 1 could pass the dominance test (*p*_1 _≥ *p_k _*for all *k*), and the OPMA would spend the entire endowment on the most productive individual (or on a subset of the most productive individuals if this designation not unique).

The utilitarian social welfare function sums the payoffs over all individuals, including the allocator. It attaches the same weight to the payoff of each and every individual and thus features other-regarding preferences more strongly than the OPMA target function:

(6) $$W_{UA}\left(\pi_{0},\pi_{1},\pi_{2},...,\pi_{n}\right)=\pi_{0}+\underset{i}{\sum }\pi_{i}.$$

When a social utilitarian allocator UA decides to serve individual *k *with the minimal endowment *m_k_*, he will consider the corresponding payoff *π_k _*= *m_k_*·*p_k _*as well as his own payoff *π*_0 _= *t*·*m_k_*·*p_k_*. The dominance test for the social utilitarian then changes to

(7) $$\left(t+1\right)\cdot m_{k}\cdot p_{k}+c>\left(t+1\right)\cdot m_{k}\cdot p_{1}.$$

Individuals that are not in position 1 (i.e. all but the most productive recipient or recipients) face a higher threshold for being served by the UA than by the OPMA. Hence, fewer potential recipients are included under the utilitarian social welfare regime than under the principle of maximizing own payoff.

### 2.2 The number maximizing allocator

An allocator maximizing the number of recipients (NMA) has the following social target function:

(8) $$W_{NMA}\left(\pi_{0},\pi_{1},\pi_{2},...,\pi_{n}\right)=\underset{i}{\sum }N_{i}\text{ with }N_{i}=\left\{\begin{array}{c} 0,\text{ if }\pi_{i}=0\\ 1,\text{ if }\pi_{i}>0 \end{array}\right).$$

This allocator first ranks the set of individuals according to increasing *m_i_*, the respective minimum ration required for a positive payoff. If two individuals need the same minimal amount, the one with higher productivity is ranked first. Let *L *be the correspondingly ranked set of individuals where *l *= 1 is the individual with the minimum *m_i_*. The number of recipients is maximized if the NMA follows the ranked individuals within *L *and allocates *m_i _*as long as the remaining endowment $R-\underset{l}{\sum }m_{l}$ is positive. Once the endowment becomes too small to serve a further individual, the allocator stops f). He will be indifferent as to how to allocate the remaining amount. To distinguish this type from the Rawlsian allocator that is discussed below, we assume that the remaining endowment is allocated along utilitarian principles, thus going to the most productive reci-pients.

### 2.3 The Rawlsian allocator with lexicographic maximin preferences

The Rawlsian allocator's (RA) preferences over two payoff distributions (*π*_0_,*π*_1_, *π*_2_,....,*π_n_*) and $\left(\pi_{0}^{*},\pi_{1}^{*},\pi_{2}^{*},...,\pi_{n}^{*}\right)$ are represented by the lexicographical comparison of the payoff vectors for all individuals 0,1,2,...,*n*, arranged in increasing order. The RA prefers distributions which maximize the payoff of the individual 0 which is worst off. If there are several individuals 0, the RA compares the payoffs of the individuals with index 1 and again prefers the allocation with the higher payoff. If these, too, are equal, he proceeds to index 3, etc. Given that, generally, not every potential recipient can be served, the RA will first maximize the number of recipients. Next, rather than increasing the ration for one individual beyond *m_i_*, he will 'save' another individual the remaining endowment permitting. Similar to the NMA allocator, the RA will thereby favor individuals with low minimal needs. But the RA differs from the NMA when it comes to the allocation of the remaining endowment. Applying Rawls' principle [2] leads to a leximin solution with respect to the payoffs, firstly, of those recipients who received at least their minimum amount and, secondly, the allocator himself. It is important here that Rawls' criterion be applied to the payoffs of the allocator and the recipients simultaneously. The allocation resulting from Rawls' criterion differs strictly from a purely egalitarian allocation, which equalizes the allocated rations without incorporating the number of recipients and without taking into account the different productivities of the potential recipients. This (naïvely) non-consequentialistic egalitarian allocation is not considered here.

## 3 Experimental design and identification of ideal types of allocators

In this section we report data from a series of experiments in which participants allocated a given amount of resources to seven potential recipients in ten different treatments. They knew that payments to themselves and to the recipients would be based on their choices in one out of the ten treatments, to be selected at random. A total of 17 experimental sessions were conducted at the Magdeburg Laboratory for Experimental Economics (MaXLab) between December 2007 and February 2008 using Urs Fischbacher's [3] software tool z-tree. 136 students from the faculties of economics and medicine participated in the experiments g). No one was permitted to participate in more than one session. The allocators included 36 economics students and 22 medical students, whereas the recipients were almost all economics students. The sessions lasted between 45 and 90 minutes. Participants received a show-up fee of €3 and payoffs depending on their choices. Average earnings were €12.65 per person. In the allocation problems, payoffs were described in experimental currency units (ECU), with 100 ECU equaling €2. We used a purely economic frame with neutrally described allocation problems in 8 sessions and a medical frame in 9 sessions, where the potential recipients were described as patients and the allocator as physician. The framing did not change during the sessions, so that no individual acted under both framings. Experimental instructions are provided in Additional File 1: Appendix B.

Eight individuals participated in each session. At the beginning of a session, we randomly chose one to be the allocator. The seven remaining participants were assigned to be recipients. Starting with session 6, we changed this aspect of the design and let all eight subjects allocate endowments among seven virtual recipients. In these sessions, only the allocators received actual payments; and they were informed that their decisions had no payoff consequences for other persons. The information about the characteristics of the recipients did not differ between the two design variants.

The total endowment of the allocator was either 1000 ECU or 1600 ECU. The allocator's par-ticipation rate in the recipients' payoffs was set at *t *= 0.2, and the fine for every individual not served at *c *= 50 ECU. From these parameter values, we can estimate the relative payoffs between the allocators and the recipients as follows: If the allocator chooses to serve all, his profit will exceed the average recipients' payoff by 40 percent since he receives one fifth of their total payoffs.

In ten treatments, each representing one allocation problem, the allocator had to decide how many ECU to give to each of the seven individuals. The characteristics of the recipients differed across the treatments; see Table 1. Their minimum thresholds *m_i _*range from 10 ECU to 1000 ECU. The last column presents the sum of all the recipients' thresholds per treatment. When given an initial endowment of 1000 ECU, the allocator could serve all individuals only in treatment 2. In all other treatments, he is forced to forego at least one recipient and pay the fine of 50 ECU. When the total endowment was increased to 1600 ECU, the allocator could in principle serve all individuals in seven out of ten treatments, thus avoiding the fine completely. The productivity factor *p_i _*ranges from 1 to 5 and indicates the extent to which a recipient benefits from his allocation. For instance, in treatment 1 a ration of 300 ECU translates into a payoff of 1200 ECU for person 1, but only 600 for person 4.

**Table 1:** The 10 allocation problems

Treatment	Person	1	2	3	4	5	6	7	$\underset{i}{\sum }m_{i}$
**1**	*m_i_*	300	50	150	50	100	300	100	1050
	*p_i_*	4	3	3	2	3	5	4	

**2**	*m_i_*	200	100	10	50	50	10	100	520
	*p_i_*	4	2	1	2	3	2	3	

**3**	*m_i_*	300	200	500	100	100	200	300	1700
	*p_i_*	4	2	1	2	3	4	1	

**4**	*m_i_*	500	100	50	50	50	100	600	1450
	*p_i_*	4	1	1	2	3	2	4	

**5**	*m_i_*	300	100	200	100	300	200	1000	2200
	*p_i_*	2	3	2	2	3	2	3	

**6**	*m_i_*	100	50	100	50	500	100	500	1400
	*p_i_*	3	3	1	2	3	2	3	

**7**	*m_i_*	200	200	200	200	200	400	400	1800
	*p_i_*	2	2	2	2	2	2	2	

**8**	*m_i_*	200	200	200	200	200	200	200	1400
	*p_i_*	1	2	3	1	3	2	2	

**9**	*m_i_*	400	50	10	10	50	600	50	1170
	*p_i_*	2	1	1	2	2	3	3	

**10**	*m_i_*	100	100	100	100	100	500	500	1500
	*p_i_*	2	2	2	2	2	3	3	

^m^_i_^: minimum ration individual *i *needs to obtain a positive payoff^

^p^_i _^productivity factor, transforming an allocated ration into a payoff for the recipient^

In a slight twist to the payoff function (2), we instructed the allocators to give each individual *i *whom they wish to serve at least *m_i_*+1 ECU in order to secure a positive payoff h). Additional File 1: Appendix A shows the optimal solutions for all types of allocators (Additional File 1 Table 1 for total endowment = 1000 ECU and Addi-tional File 1 Table 2 for total endowment = 1600 ECU).

**Table 2:** The average treatment payoffs of the allocators and their recipients for the four ideal types of social orientation (in parentheses: in percentage of the OPMA type)

	Allocator's payoff *π*_0_		Sum of recipients' Payoffs $\underset{i}{\sum }\pi_{i}$		Total payoff $\pi_{0}+\underset{i}{\sum }\pi_{i}$	
**Type**	***R *= 1000**	***R *= 1600**	***R *= 1000**	***R *= 1600**	***R *= 1000**	***R *= 1600**

**UA**	443.24 (86.8)	881.24 (92.7)	3391.20 (112.0)	5431.20 (107.0)	3834.44 (108.4)	6312.44 (104.7)

**OPMA**	510.42 (100)	950.42 (100)	3027.10 (100)	5077.10 (100)	3537.52 (100)	6027.52 (100)

**NMA**	505.30 (99.0)	918.04 (96.6)	2926.50 (96.7)	4665.20 (91.9)	3431.80 (97.0)	5583.24 (92.6)

**RA**	433.03 (84.8)	781.76 (82.3)	2565.13 (84.7)	3983.81 (78.5)	2998.15 (84.8)	4765.57 (79.1)

^UA: utilitarian allocator, OPMA: own payoff-maximizing allocator, NMA: number maximizing allocator, RA: Rawlsian allocator^

Table 2 shows the average payoffs for the ideal types of allocators and their recipients in every treatment. Compared to the utilitarian type, the OPMA reduces the total payoff. The NMA, and particularly the RA, reduce total payoff even further. They both choose a lower payoff for themselves than the OPMA does. Moreover, they allocate lower average payoffs to the recipients than the OPMA. These results, of course, reflect the target functions of the NMA and the RA, because both types give primary consideration to maximizing the number of recipients and consider the recipients' payoffs only as a secondary criterion.

When the total endowment is higher, allocator and recipient payoffs differ more - in percent - under number maximizing or Rawlsian social orientation than under the OPMA principle. This effect is reversed for the utilitarian type: Here the percentage difference between the allocator's payoffs and the total payoff is bigger if *R *= 1000 ECU, but smaller for the sum of the recipients' payoffs. Being a utilitarian (and maximizing total payoff) rather than being selfish is more "costly" to the allocator if the available amount to be distributed is smaller, i.e. when scarcity is more severe. For the other types, the costs of being utilitarian as compared to being selfish are higher when the shadow price of the resource constraint increases.

The 58 allocators made a total of 3,948 allocation decisions i. Table 3 provides information on the number of treatments, allocators and observations in the two framings, for high and low budgets, real and virtual recipients, and the allocator's profession. A treatment is defined as a decision problem in which a given endowment is allocated among 7 potential recipients. A treatment thus provides 7 observations of allocations. With ten treatments in a session, each allocator takes 70 decisions in total.

**Table 3:** Number of treatments, allocators, and observations

Framing	Budget	Recipients	No. of treatments	No. of allocators		No. of observations	
				
				Economists	Physicians	Economists	Physicians
Medical	high	real	0	0	0	0	0
		virtual	134	7	8	378	560
	
	low	real	60	0	6	0	420
		virtual	80	8	0	560	0

Neutral	high	real	0	0	0	0	0
		virtual	160	8	8	560	560
	
	low	real	50	5	0	350	0
		virtual	80	8	0	560	0

**Total**			**564**	**36**	**22**	**2408**	**1540**

## 4 Results

### 4.1 Classification of allocator

Based on their actual choices, we classified the allocator-subjects according to their proximity to one of the four ideal types described above using a variance test j). An allocator was classified as belonging to one type if this mean Euclidian distance from the respective optimal choice was significantly smaller than his mean Euclidian distance from the optimal choices of the three alternatives. Table 4 shows the classification results for both the economic and the medical settings. In addition to 'pure' types, we also observe individuals which are in between two types. If these tests were inconclusive for an allocator - so neither significantly close to one type nor in between two types - (at the 10 percent significance level), this individual was not classified.

**Table 4:** The classification of the allocators in the two settings

	Economists			Physicians		
**Framing** **Type**	**neutral**	**medical**	**Total**	**neutral**	**medical**	**total**

**UA**	**0**	**0**	**0**	**0**	**0**	**0**
UA/OPMA	0	1	1	0	0	0
**OPMA**	**6**	**1**	**7**	**1**	**1**	**2**
OPMA/NMA	1	2	3	0	1	1
**NMA**	**10**	**6**	**16**	**2**	**4**	**6**
NMA/RA	0	1	1	2	3	5
**RA**	**2**	**1**	**3**	**3**	**4**	**7**

**Not classified**	**2**	**3**	**5**	**0**	**1**	**1**

**Total**	**21**	**15**	**36**	**8**	**14**	**22**

^UA: utilitarian allocator, OPMA: own payoff-maximizing allocator, NMA: number maximizing allocator, RA: Rawlsian allocator^

Not one of the 58 allocators was classified as being a social utilitarian who maximizes the total payoff. Among the economists, 19 percent were classified as OPMA, compared to 9 percent among the physicians. At 44 percent, the share of NMA among economists is higher than that among physicians by a factor of 1.6. Only 3 of the 36 economists were classified as RA, while as many as 7 out of 22 physicians appear to lean towards Rawlsian leximin allocations. The mixed types confirm this tendency: 5 physicians and only 1 economist were classified as the mixed NMA/RA type. Unclassified allocators made up around 14 percent among econo-mists and only 5 percent among physicians.

### 4.2 Framing and professional effects

In this section, we want to shed light on the effects of the medical and neutral framing as well as on possible differences in the choices made by economists and physicians. Table 5 shows the mean Euclidean distances of the decisions made by the three allocator types in the two professional populations, based on the allocated payoffs including the allocator's. While economists lean towards maximizing their own payoff, the physicians are closest to the allocator type that maximizes the number of recipients.

**Table 5:** Euclidian distance from types of allocators; economists and physicians

	Economists		Physicians	
**Framing** **Type**	**neutral**	**medical**	**neutral**	**medical**

**OPMA**	444	643	789	612

**NMA**	399	502	552	462

**RA**	622	682	569	528

^OPMA: own payoff-maximizing allocator, NMA: number maximizing allocator, RA: Rawlsian allocator^

Regarding framing, the results suggest that economists move further away from the allocator types - most accentuated in the case of the OPMA - when the setting is medical as compared to the neutral, purely economic framing. By contrast, physicians are closer to one of the three types when the framing is medical. Thus, it appears that the classification becomes clearer when the setting corresponds to the allocator's own professional background. When the setting is unfamiliar, the categories of the classification system prove less powerful. This seems to indicate that "professional norms" guide these decisions. This holds even for the OP-MA, for the corresponding motivations become more forceful in the setting in which it is considered legitimate to maximize one's own payoff.

Table 6 gives the results of variance tests for the professional and framing effects described above. The last row shows that physicians are significantly further away from the OPMA payoff than economists. The opposite goes for the Rawlsian types. The framing effects are surprising, since they work in different directions for economists and physicians. When the setting is medical, economists allocate in less own-payoff maximizing ways, while physicians move towards payoff maximization. Economists seem to get cold feet in the medical setting and move away from their professional focus on maximizing a given objective function. It holds well for the physicians, too, that their professional norms emerge more clearly in the setting familiar to them k).

**Table 6:** Framing and professional effects (relative squared Euclidian distance to the types of allocator) - results from a variance test

Type	OPMA	NMA	RA
**Faculty**	Framing effect (medical vs. neutral setting)		

**Economists**	1.45***	1.26*	1.10
**Physicians**	(1/1.29)**	(1/1.20)	(1/1.08)

	Faculty effect (physicians vs. economists)		

	1.28**	1.12	(1/1.19)**

^OPMA: own payoff-maximizing allocator, NMA: number maximizing allocator, RA: Rawlsian allocator^

***, (**), [*] significant at the 99% (95%) [90%] confidence level, resp.

### 4.3 Efficiency costs

The efficiency cost of an allocator's choices corresponds to the deviation from the social utilitarian welfare $\pi_{0}+\underset{i}{\sum }\pi_{i}$. Table 7 shows these costs by profession and framing. While framing effects are more or less absent, the difference between economists and physicians is considerable and statistically significant. While the economists' choices lead to an efficiency cost of between 9 and 12 percent, the choices made by the physicians involve an efficiency loss of 16 to 20 percent. The size of the efficiency costs in percentage terms appears not to depend on the total endowment.

**Table 7:** Social utilitarian welfare of the allocators' choices and their own profit (in parentheses: in percentage of the respective reference)

		Economists		Physicians	
	**Framing** **Reference**	**neutral**	**medical**	**neutral**	**medical**

	**UA**^a)^	Social utilitarian welfare: $\pi_{0}+\underset{i}{\sum }\pi_{i}$			

*R *= 1000	3834.44 (100)	3375.97 (88.0)	3417.36 (89.1)	--- (---)	3087.71 (80.5)
*R *= 1600	6312.44 (100)	5695.27 (90.2)	5787.23 (91.7)	5289.35 (83.8)	5255.80 (83.3)

	**OPMA**^a)^	Own profit *π*_0_			

*R *= 1000	510.42 (100)	456.57 (89.4)	460.71 (90.3)	--- (---)	422.26 (82.7)
*R *= 1600	950.42 (100)	908.07 (95.5)	910.53 (95.8)	838.85 (88.3)	838.99 (88.3)

^UA: utilitarian allocator, OPMA: own payoff-maximizing allocatora) ^for the values see Table 2

Interestingly, although we find many OPMA, the second part of Table 7 indicates that the average willingness to sacrifice one's own profits to pursue other goals is large. It compares the allocators' own payoffs to those of an ideal-type OPMA. Economists choose a payoff that is between 4.5 percent and 10.6 percent lower than that of the OPMA. For physicians, the payoff is as much as 11.7 percent to 17.3 percent lower. The allocators' sacrifice of their own profits decreases when total endowment increases. As with the efficiency costs, framing effects are absent and professional effects are statistically significant regarding the chosen amount of own profit.

## 5 Who is served and how much do they receive? Hypotheses and tests

The way an allocator distributes the endowment depends on his target function, the budget and the characteristics of the potential recipients. As the latter parameters are the same for all allocators, differences among them will arise due to differences in their target functions.

### 5.1 The determinants of a positive payoff

Let us first consider the OPMA. His criterion for serving an individual is the dominance test (5), which can be rewritten as

(9) $$p_{k}+\frac{c}{t\cdot m_{k}}>\;p_{1}.$$

It follows that the OPMA is more likely to choose individuals who are very productive or need only a small minimum ration.

An NMA and an RA will not, as a first criterion, consider the individuals' productivity when deciding whom to serve. The decisive parameter in their first move is the individuals' minimum need. They will first choose individuals with a small minimum need, allowing them to maximize the number of recipients with positive payoffs. If, for example, there are two individuals with the same minimum need but different productivities and only one of them can be served, we assume that the NMA then allocates the minimum amount to the individ-ual with the higher productivity (and the rest to the served recipient with the highest productivity). The RA will also choose this individual, but allocate the remaining amount according to the leximin criterion. The distributive problems often feature several recipients with the same minimum amount but different productivities. If not all of them can be served, productivity will play a role. We therefore also expect productivity to have a small influence on the probability of being served by these types.

Under certain parameter constellations (different from those in our experiments), the own-payoff maximizing selection of recipients could even equal that of the other allocators. If the participation factor *t *were very low or the fine *c *were very high, every individual would pass the dominance test. In this case, an OPMA will also maximize the number of recipients with positive payoffs and serve the same individuals as the other allocators.

We can thus derive the following propositions concerning the allocation of positive rations to individuals:

*Proposition set 1: The OPMA, the NMA, and the RA are more likely to serve an individual whose minimum need is low. Only the OPMA has a strong concern regarding an individual's productivity. His willingness to allocate a positive ration increases the more productive the potential recipient is*.

In order to test proposition set 1, we ran a logit regression that exploits the panel structure of the data. We applied a random effects model to account for the possibility of specific correlation between the error terms of an allocator's choices. Then we included dummy variables for the treatments, since the shadow price of the total endowment depends on the sum of the minimum thresholds, which differs from treatment to treatment. Treatment 2 has the lowest sum $\underset{i}{\sum }m_{i}=520$ ECU and served as benchmark treatment. Total endowment is included with a dummy variable which takes on the value 1 for *R *= 1600 ECU and 0 for *R *= 1000 ECU. Moreover, absolute and slope dummies for the different types were incorporated, with the unclassified allocators serving as benchmark.

For the potential recipients, the mean probability of a positive payoff was 75.3 percent. Table 8 presents the results of the logit model. The likelihood ratio test for rho = 0 indicates that the joint hypothesis of zero slope coefficient can be rejected.

**Table 8:** Explaining the probability of positive payoffs: a logit model

Variables	Coefficient	**Std. err**.	Variable	Coefficient	**Std. err**.
Constant	-0.790	0.543	OPMA	0.024	0.652
treatment_1	-2.557***	0.313	OPMA ***^.^***NMA	0.457	0.643
treatment_3	-1.376***	0.306	NMA	1.149**	0.554
treatment_4	-1.078***	0.295	NMA***^.^***RA	1.748***	0.556
treatment_5	-1.326***	0.297	RA	2.252***	0.708
treatment_6	-0.776***	0.302			
treatment_7	-1.002***	0.290	Minimum need	-0.007***	0.001
treatment_8	-1.377***	0.289	OPMA ***^.^***minimum need	0.001	0.001
treatment_9	0.039	0.315	NMA ***^.^***minimum need	-0.002***	0.001
treatment_10	-0.920***	0.299	RA ***^.^***minimum need	0.000	0.001
					
Economic framing	-0.349	0.273	Productivity	1.314***	0.117
1 allocator only	0.398	0.380	OPMA***^.^***Productivity	0.282	0.193
Endowment	1.568***	0.312	NMA ***^.^***productivity	0.422***	0.166
			RA ***^.^***productivity	0.043	0.224

Number of observations: 3,948			Number of groups: 58		

Pseudo R^2^: 0.26			Rho:	0.188***	0.042

^UA: utilitarian allocator, OPMA: own payoff-maximizing allocator, NMA: number maximizing allocator, RA: Rawlsian allocator^

***, (**), [*] significant at the 99% (95%) [90%] confidence level, resp.

Regarding the treatment effect on the probability of being served, the coefficient shows the expected sign. As the resources were always scarcer than in treatment 2, the probability of a positive payoff was significantly lower in those treatments.

In the neutral framing of the allocation problem fewer potential recipients were served than in the medical setting, though the difference is not significant. The dummy for sessions where only one individual acted as allocator is positive, indicating that the presence of actual recipients in the laboratory positively affects the allocator's willingness to serve them. This result, however, is not significant. The endowment dummy shows the expected sign. When the endowment rises to 1600 ECU, significantly more potential recipients are served.

The intercept dummies differentiate between pure and mixed types of allocators. NMA, RA, and their mixed type served more recipients than OPMA on average. The probability of a positive payoff falls significantly with an increase in a potential recipient's minimum threshold. This effect is significant and stronger for NMA, but not for OPMA and RA.

Finally, the productivity of potential recipients has a large effect on the probability of being served. The effects are significant and larger for NMA, but not for OPMA.

### 5.2 Explaining the conditioned positive allocation

The second choice refers to the size of the allocated ration, conditional on the payoff being positive. The experimental design implies that the ration must be higher than the minimum threshold. Therefore, explaining the conditioned positive allocation means explaining the extra ration *r_i_*- *m_i_*. We already know that OPMA allocates the minimum ration to all individuals he chooses to serve, except for the most productive, who receives the entire remaining endowment. A general relationship between the allocated extra ration *r_i_*- *m_i _*and a potential recipient's productivity cannot be derived, as the extra ration will be positive for the most productive individual only. Still, a negative correlation can be excluded.

The allocator maximizing the number of recipients will, in a first stage, be indifferent as to who receives the rest. We assume that, in a second stage, he wants to maximize his profit. Like the selfish distributor, he will allocate the remaining endowment to the most productive recipients.

The Rawlsian allocator will try to improve the payoff of those with a low initial level of *p_i_*·*m_i_*. Hence, the extra ration *r_i_*- *m_i _*received should be negatively correlated with *p_i_*·*m_i_*.

This comparative statics analysis leads to

*Proposition set 2: The extra rations allocated by OPMA and NMA are positively related to the recipients' productivity. RA will increase a recipient's ration whose initial payoff (*p_i_·m_i_*) is low*.

The payoff was positive in 77.7 percent of the decisions, providing 3,068 observations. Reci-pients received an average extra ration of 64.04 ECU. Table 9 presents the results of a random effects OLS estimation with the extra ration as the dependent variable.

**Table 9:** Explaining the extra ration *r_i_*-*m*_*i*_of recipients with positive payoffs (OLS with random effects)

Variables	Coefficient	**Std. err**.
Constant	10.99	18.61
OPMA	-233.52***	29.14
OPMA ***^.^***NMA	-105.82***	23.24
NMA	-122.39***	23.40
NMA***^.^***RA	-102.11***	19.96
RA	-81.79***	20.38
Endowment	33.20***	9.62
Minimum need	-0.01	0.02
Productivity	60.18***	4.61
OPMA ***^.^***productivity	53.82***	8.72
NMA ***^.^***productivity	2.78	6.46
RA ***^.^***productivity ***^.^***minimum need	-0.08***	0.01

Number of observations = 2,973 Number of Groups = 58		

R^2^: within = 0.169 R^2^: between = 0.279 R^2^: overall = 0.175		

^OPMA: own payoff-maximizing allocator, NMA: number maximizing allocator, RA: Rawlsian allocator^

***, (**), [*] significant at the 99% (95%) [90%] confidence level, resp.

The sign of the coefficients for the different types' intercept dummies can be explained as follows. As OPMA and NMA choose an extra ration of zero more often than the other types (see the last section), their allocated extra rations are on average lower. When the endowment is larger, the average extra ration increases, which is not necessarily expected.

The signs of the slope dummies for the allocator types are also as expected. In the case of OPMA and NMA, the allocated extra rations grow with increasing recipient productivity, though not significantly for NMA. The RA's extra rations are negatively correlated with *p_i_*·*m_i_*, as we hypothesized.

### 5.3 Discussion of the variance in the behavior

Clearly, theories of inequality aversion ([4-8], and [1]) seem relevant for the allocation decisions we study in our experiment. We use the Fehr-Schmidt model [4] to explain how these theories shape the predictions for our pure social choice types and increase the variance in the data. In this approach (Bolton-Ockenfels [5] is similar), the participants obtain positive utility from decreasing inequality. The theory is based on the idea that, due to social comparisons, one's utility decreases either if one has a lower payoff than others (envy) or a higher payoff than others (altruism). The relative importance of these two aspects of social orientation can be adjusted by the choice of positive parameters a_0 _and b_0_, respectively. The measure for lower and higher payoffs is given by the sum of the distance of the others' payoff to one's own. Finally, the utility function is additively separable into utility derived from one's own payoff and disutility derived from social comparisons (i.e. can be split into an envy and an altruism part):

(10) $$W_{FS}\left(\pi_{1},...,\pi_{n}\right)=\pi_{0}-\frac{a_{0}}{N-1}\underset{i}{\sum }\max\left(\left\{\pi_{i}-\pi_{0},0\right)\right\}-\frac{b_{0}}{N-1}\underset{i}{\sum }\max\left(\left\{\pi_{0}-\pi_{i},0\right)\right\}$$

If we changed the utility functions of the allocators in our experiment accordingly, we would obviously get deviations from the pure types we characterized in the previous sections. Applying the Fehr-Schmidt utility function to a selfish allocator produces deviations towards the Rawlsian type, who favors more equal payoffs even for small values of *b*_0_. Applying the Fehr-Schmidt utility function to the Rawlsian type, we would get towards the selfish allocator. Depending on the parameters, the deviations would vary according to the personal characteristics of the decision maker. The variance in our data might be partly explained by mixed types of social orientation. Since we are interested in the pure social choice types and did not use target functions with two parameters, we accept a less good fit of our data with the theoretical choice predic-tion.

## 6 Conclusion

We started our analysis of decision behavior by applying different distributive principles that reflect general social orientation. We determined allocations resulting from selfish payoff maximization, allocations that maximize a utilitarian sum of payoffs including that of the allocator, allocations that maximize the number of treated patients, and Rawlsian allocations.

There is a vast health economics literature that deals with the effects of patient-related factors (such as age, social role, and health-related lifestyle) and of treatment characteristics (the duration of the intervention effects and severity of an illness prior to intervention) on the allocation decisions made by physicians (see [9] for an overview). This experiment implements two abstract characteristics of patients (minimum need and the productivity of treatment) and the clearable resource (time or money) and dissociates from specific treatment conditions and patient characteristics.

We did not identify any social utilitarians among the 58 allocators. Correspondingly, the social costs of the observed choices are substantial, ranging from 10 percent for economists to 20 percent for physicians. On the other hand, the allocators do not appear to maximize their own payoff. The willingness to forego own payoff is considerable, around 5 to 17 percent of the maximum possible profit. The amount is smaller for economists, but both professional groups sacrifice an amount of money for purposes other than their own payoff. The sacrificed percentage becomes smaller if the endowment increases.

One in five economists is an own payoff maximizer, whereas only one in eleven physicians can be classified in this way. By comparison, one in three physicians is a Rawlsian, compared to only one in twelve economists. Thus, the distributive norms held by different professions appear to have an influence on people's decisions. Furthermore, the economists are sensitive to framing effects. They are closer to own payoff-maximizing behavior in the neutral framing than in the medical setting. Surprisingly, physicians are closer to own-payoff maximization in the medical setting, though their behavior is generally less strongly affected by framing.

Another set of results focuses on monotonicity relations, which are important theoretical properties of many allocation rules. How do the decision makers react to an increase in the amount of the available resource? We find that if resources are scarcer, the probability of a positive payoff for the recipients is significantly smaller for most of the treatments as fewer potential recipients are served. This is in line with normative properties of resource monotonicity. For example, this axiom, typically applied to distributive problems in normative bargaining theory (cf. [10]), requires that an increase in the resource not make anybody worse off. Although we did not analyze this property on an individual basis, the data on the probability of being served and on the number of recipients with positive payoff do in fact show a reaction in the required direction. This observation holds for all the allocators and can therefore be interpreted as a general property of revealed social orientation.

Two other monotonicity relations are fundamental due to the structure of the decision prob-lems: reactions to differing minimum needs and to differing productivities of the potential recipients. A smaller minimum need should in general lead to a higher probability of being served. Again, we observe the expected tendency for all types of allocators. However, the effect is strongest for the allocator maximizing the number of recipients. The effect of higher productivity on the probability of receiving a positive payoff is positive, as one would expect. Surprisingly, this effect is strongest in the case of the number maximizing allocator. One explanation stems from the specific design of the allocation problems: Conflicts can arise in deciding on the last individual to include in the group of actual recipients. If there are two potential recipients with the same minimum need but with different productivities and only one can be served, which one should it be? The data confirm that, in such a conflict, productivity is a decisive property for the allocator maximizing the number of recipients.

## Competing interests

The authors declare that they have no competing interests.

## Authors' contributions

MA, SF, and BV carried out the analysis and wrote the paper together. All three authors read and approved the final manuscript.

## Supplementary Material

Additional File 1 **Supporting information**. Contains additional data detailing experimental design and sample instructions for the experiment. Click here for file
