# Supplementary material for: Which patients do I treat? An experimental study with economists and physicians
Source: Health Econ Rev. 2012 Jan 5;2:1. doi: 10.1186/2191-1991-2-1 (PMC3402930; doi:10.1186/2191-1991-2-1)
Supplement: Additional File 1 — Supporting information. Contains additional data detailing experimental design and sample instructions for the experiment. [file 2191-1991-2-1-S1.DOC]

**Appendix A: Optimal choices for the types of allocator**

**Table A1: Optimal payoffs when total endowment is 1000 ECU; by types of allocator^a)^**

| **Treatment** | **Type** | **Allocator** | **Recipient** | | | | | | |
| --- | --- | --- | --- | --- | --- | --- | --- | --- | --- |
|  |  | **0** | **1** | **2** | **3** | **4** | **5** | **6** | **7** |
| **1** | UA | 700 | 0 | 0 | 0 | 0 | 0 | 5000 | 0 |
|  | OPMA | 788.4 | 0 | 153 | 0 | 102 | 303 | 3480 | 404 |
|  | NMA | 778.2 | 1204 | 153 | 0 | 102 | 303 | 1975 | 404 |
|  | RA | 729.4 | 1204 | 267 | 0 | 214 | 303 | 1505 | 404 |
| **2** | UA | 589 | 3912 | 0 | 11 | 0 | 0 | 22 | 0 |
|  | OPMA | 697.8 | 2696 | 202 | 11 | 102 | 153 | 22 | 303 |
|  | NMA | 697.8 | 2696 | 202 | 11 | 102 | 153 | 22 | 303 |
|  | RA | 467.8 | 804 | 246.4 | 246.4 | 246.4 | 246.4 | 246.4 | 303 |
| **3** | UA | 550 | 2000 | 0 | 0 | 0 | 0 | 2000 | 0 |
|  | OPMA | 589.4 | 2388 | 0 | 0 | 202 | 303 | 804 | 0 |
|  | NMA | 559 | 1584 | 402 | 0 | 202 | 303 | 804 | 0 |
|  | RA | 524.6 | 1204 | 402 | 0 | 356.4 | 356.4 | 804 | 0 |
| **4** | UA ^b)^ | 500 | 4000 | 0 | 0 | 0 | 0 | 0 | 0 |
|  | OPMA ^b)^ | 598.4 | 2984 | 0 | 51 | 102 | 153 | 202 | 0 |
|  | NMA ^b)^ | 587.8 | 2580 | 101 | 51 | 102 | 153 | 202 | 0 |
|  | RA | 505.1 | 2004 | 138.8 | 138.8 | 138.8 | 153 | 202 | 0 |
| **5** | UA | 350 | 0 | 1500 | 0 | 0 | 1500 | 0 | 0 |
|  | OPMA | 399.4 | 0 | 588 | 402 | 202 | 903 | 402 | 0 |
|  | NMA | 399.4 | 0 | 588 | 402 | 202 | 903 | 402 | 0 |
|  | RA | 384 | 0 | 356.4 | 402 | 356.4 | 903 | 402 | 0 |
| **6** | UA ^b)^ | 400 | 1344 | 153 | 0 | 0 | 1503 | 0 | 0 |
|  | OPMA ^b)^ | 479.2 | 585 | 153 | 101 | 102 | 1503 | 202 | 0 |
|  | NMA ^b)^ | 479.2 | 585 | 153 | 101 | 102 | 1503 | 202 | 0 |
|  | RA | 448.8 | 303 | 162 | 162 | 162 | 1503 | 202 | 0 |
| **7** | UA ^c)^ | 250 | 500 | 500 | 500 | 500 | 0 | 0 | 0 |
|  | OPMA ^c)^ | 250 | 794 | 402 | 402 | 402 | 0 | 0 | 0 |
|  | NMA ^c)^ | 250 | 794 | 402 | 402 | 402 | 0 | 0 | 0 |
|  | RA ^c)^ | 250 | 500 | 500 | 500 | 500 | 0 | 0 | 0 |
| **8** | UA | 350 | 0 | 0 | 1500 | 0 | 1500 | 0 | 0 |
|  | OPMA ^c)^ | 369.6 | 0 | 0 | 1191 | 0 | 603 | 402 | 402 |
|  | NMA ^c)^ | 369.6 | 0 | 0 | 1191 | 0 | 603 | 402 | 402 |
|  | RA ^c)^ | 330.4 | 0 | 598 | 603 | 0 | 603 | 0 | 598 |
| **9** | UA | 443.4 | 0 | 0 | 11 | 22 | 0 | 1803 | 1131 |
|  | OPMA | 512.8 | 0 | 51 | 11 | 22 | 102 | 2475 | 153 |
|  | NMA | 512.8 | 0 | 51 | 11 | 22 | 102 | 2475 | 153 |
|  | RA | 290.1 | 802 | 179.7 | 179.7 | 179.7 | 179.7 | 0 | 179.7 |
| **10** | UA ^a)^ | 300 | 0 | 0 | 0 | 0 | 0 | 0 | 3000 |
|  | OPMA ^d)^ | 419.2 | 0 | 202 | 202 | 202 | 202 | 0 | 1788 |
|  | NMA ^d)^ | 419.2 | 0 | 202 | 202 | 202 | 202 | 0 | 1788 |
|  | RA ^d)^ | 400.2 | 0 | 249.5 | 249.5 | 249.5 | 249.5 | 0 | 1503 |

UA: utilitarian allocator, OPMA: own payoff-maximizing allocator, NMA: number maximizing allocator, RA: Rawlsian allocator

^a)^ In some treatments, multiple optimal solutions exist for all types (e.g. when several of the recipients that are being served have the maximal productivity). This was considered in the classification (see footnote 9). The number of alternative solutions is given in footnotes b to d.

^b)^ There is one alternative solution to the optimal choice given here.

^c)^ There are two alternative solutions to the optimal choice given here.

^d)^ There are nine alternative solutions to the optimal choice given here.

Note: An example for the alternative solution for UA in treatment 4: Individual 0 (the allocator) receives a payoff of 500 ECU, individuals 1 to 7 (recipients) receive 0, individual 8 (recipient) receives 4000 ECU.

**Table A2: Optimal payoffs when total endowment is 1600 ECU; by type of allocator**a)

| **Treatment** | **Type** | **Allocator** | **Recipient** | | | | | | |
| --- | --- | --- | --- | --- | --- | --- | --- | --- | --- |
|  |  | **0** | **1** | **2** | **3** | **4** | **5** | **6** | **7** |
| **1** | UA | 1300 | 0 | 0 | 0 | 0 | 0 | 8000 | 0 |
|  | OPMA | 1388.4 | 0 | 153 | 0 | 102 | 303 | 6480 | 404 |
|  | NMA | 1367.8 | 1204 | 153 | 453 | 102 | 303 | 4220 | 404 |
|  | RA | 1112.1 | 1204 | 570.3 | 570.3 | 570.3 | 570.3 | 1505 | 570.3 |
| **2** | UA | 1069 | 6312 | 0 | 11 | 0 | 0 | 22 | 0 |
|  | OPMA | 1177.8 | 5096 | 202 | 11 | 102 | 153 | 22 | 303 |
|  | NMA | 1177.8 | 5096 | 202 | 11 | 102 | 153 | 22 | 303 |
|  | RA | 690.9 | 804 | 441.8 | 441.8 | 441.8 | 441.8 | 441.8 | 441.8 |
| **3** | UA | 1030 | 3200 | 0 | 0 | 0 | 0 | 3200 | 0 |
|  | OPMA | 1069.4 | 4788 | 0 | 0 | 202 | 303 | 804 | 0 |
|  | NMA | 908.4 | 2780 | 402 | 0 | 202 | 303 | 804 | 301 |
|  | RA | 728.1 | 1204 | 470.6 | 0 | 470.6 | 470.6 | 804 | 470.6 |
| **4** | UA | 1030 | 3200 | 0 | 0 | 0 | 0 | 0 | 3200 |
|  | OPMA | 1128.4 | 2980 | 0 | 51 | 102 | 153 | 202 | 2404 |
|  | NMA | 1117.8 | 2576 | 101 | 51 | 102 | 153 | 202 | 2404 |
|  | RA | 1035.6 | 2004 | 138.4 | 138.4 | 138.4 | 153 | 202 | 2404 |
| **5** | UA | 760 | 0 | 894 | 0 | 0 | 903 | 0 | 3003 |
|  | OPMA | 789.8 | 0 | 591 | 0 | 202 | 903 | 0 | 3003 |
|  | NMA | 749.2 | 602 | 1485 | 402 | 202 | 903 | 402 | 0 |
|  | RA | 686.5 | 602 | 544.4 | 544.4 | 544.4 | 903 | 544.4 | 0 |
| **6** | UA | 810 | 750 | 750 | 0 | 0 | 1650 | 0 | 1650 |
|  | OPMA | 889.2 | 882 | 153 | 101 | 102 | 1503 | 202 | 1503 |
|  | NMA | 889.2 | 882 | 153 | 101 | 102 | 1503 | 202 | 1503 |
|  | RA | 832.2 | 303 | 213 | 213 | 213 | 1503 | 213 | 1503 |
| **7** | UA | 590 | 790 | 402 | 402 | 402 | 402 | 802 | 0 |
|  | OPMA ^b)^ | 590 | 790 | 402 | 402 | 402 | 402 | 802 | 0 |
|  | NMA ^b)^ | 590 | 790 | 402 | 402 | 402 | 402 | 802 | 0 |
|  | RA ^b)^ | 590 | 479.6 | 479.6 | 479.6 | 479.6 | 479.6 | 802 | 0 |
| **8** | UA | 710 | 0 | 0 | 2400 | 0 | 2400 | 0 | 0 |
|  | OPMA | 739.4 | 0 | 402 | 2388 | 0 | 603 | 402 | 402 |
|  | NMA | 678.6 | 201 | 402 | 1182 | 201 | 603 | 402 | 402 |
|  | RA | 601.4 | 297.5 | 402 | 603 | 297.5 | 603 | 402 | 402 |
| **9** | UA | 803.4 | 0 | 0 | 11 | 22 | 0 | 2334 | 2400 |
|  | OPMA | 872.8 | 0 | 51 | 11 | 22 | 102 | 4275 | 153 |
|  | NMA | 842.6 | 802 | 51 | 11 | 22 | 102 | 3072 | 153 |
|  | RA | 700.4 | 802 | 179.4 | 179.4 | 179.4 | 179.4 | 1803 | 179.4 |
| **10** | UA | 710 | 0 | 0 | 0 | 0 | 0 | 2400 | 2400 |
|  | OPMA | 859 | 202 | 202 | 202 | 202 | 202 | 1782 | 1503 |
|  | NMA | 859 | 202 | 202 | 202 | 202 | 202 | 1782 | 1503 |
|  | RA | 840.4 | 239.2 | 239.2 | 239.2 | 239.2 | 239.2 | 1503 | 1503 |

UA: utilitarian allocator, OPMA: own payoff-maximizing allocator, NMA: number maximizing allocator, RA: Rawlsian allocator

^a)^ In some treatments, multiple optimal solutions exist for all types (e.g. when several of the recipients that are being served have the maximal productivity). This was considered in the classification (see footnote 9). The number of alternative solutions is given in footnotes b to d.

^b)^ There is one alternative solution to the optimal choice given here.

Note: An example for the alternative solution for RA in treatment 7: Individual 0 (the allocator) receives a payoff of 500 ECU, individuals 1 to 5 receive 479.6 ECU each, individual 6 receives 0, individual 7 receives 802 ECU.

**Appendix B: Sample Instructions**

**Instructions for the role of a physician**

Thank you for participating in this experiment. Please read the instructions carefully. With luck you can earn money.

These experiments study treatment decision physicians make regarding their patients. The role of physician or patient, randomly chosen by your seat number, remains fixed throughout the experiment.

You have the role of a physician. You own 1000 units of treatment time, which allows you to treat 7 patients at most. For simplicity, the treatment success of a patient will only depend on the amount of time you spend on him/her. We denote the treatment time of patient *i* by $r_{i}$. The patient’s utility is reflected by a monetary payment $\pi_{i}$.

First, each of the seven patients is characterized by two properties: Each patient *i* ($i=1,...,7$) needs a certain individual minimum quantity $m_{i}$ of treatment time for the successful completion of the treatment. If the patient receives less than this minimum quantity, the treatment is not successful. In the experiment, this is reflected by a payoff of zero for the patient ($\pi_{i}=0$).

Second, the patients will respond differently to the treatment. The experiment reflects this as follows: each patient has an individual productivity factor $p_{i}$ which describes how much utility in ECU (Experimental Currency Units) he can achieve based on the allocated quantity of treatment time; in medical terms the productivity factor can be seen as a prognosis on the success of the treatment. For each time unit of treatment, a patient’s productivity is the same; the time employed for the patient $r_{i}$ is multiplied by the individual productivity factor $p_{i}$. The productivity factor differs only between patients.

Once you have allocated all 1000 units of treatment time, with patient *i* receiving $r_{i}$ units, you can determine the consequences of your allocation. The experiment reflects this by payoffs not only to the patient, but also to you personally. The patients’ payoffs as well as your own payoff are in ECU.

Both patient and physician are informed of the experiment’s design. A patient is only informed of his own characteristics (productivity factor, minimum quantity, and payoff). The physician is informed of the characteristics of all patients.

Payoffs in ECU

1. Each patient to whom you allocated more than the minimum quantity of treatment time receives an ECU payoff equal to $\pi_{i}=r_{i}\cdot p_{i}$
2. Patients to whom you allocated an amount of time less than or equal to the required minimum will achieve zero utility (no healing); his payoff in ECU will be $\pi_{i}=0$. Hence you have to allocate at least one unit more than the minimum quantity of treatment time to achieve a positive payoff for the patient.
3. The physician (i.e. you) receives a payoff which is proportional to the utility that the patient experiences from the treatment (provided the patient receives at least the quantity $m_{i}+1$ of treatment time): your payoff is proportional to the sum $\pi_{1}+...+\pi_{7}$. The proportionality factor is 20%. For each patient who is not healed your payoff is reduced: for each patient who receives less than the minimum quantity of treatment time, 50 ECU is deducted from your payoff.

To help you make your decisions, a calculator in Excel is available to you. If you enter the productivity factor, the minimum quantity, and your planned choice, you will see the payoffs of all patients as well as your own payoff.

You have to solve allocation problems in different rounds. The number assigned to patients may change, so it is not possible for you to be able to identify a patient across different rounds. After the completion of all rounds, one round is chosen at random and the payoffs paid out in Euros. A ball will be drawn from an urn containing as many balls as rounds are played. The number on the drawn ball indicates the round that will be paid out. Payouts are made based on an exchange rate of 100 ECU = 2 Euros.

Now, please allocate your total endowment of treatment time to the patients of the first round, whose productivity factors and minimum quantity of treatment time you see on the screen.
